# The Influence of Social Media on the Decision of Rhinoplasty Among Adults in the Western and Southern Regions of Saudi Arabia: A Comparative Cross-Sectional Study

**DOI:** 10.7759/cureus.40137

**Published:** 2023-06-08

**Authors:** Medhat Taha, Hassan Ali A AlZubaidi, Ibrahim H Alkhairy, Abdulaziz Mohammed O Alsokani, Hassan Alsuhabi, Ahmed Ali A Alrezqi, Abdullah Aqeel S Alrashdi, Abdulwahab Abdulaziz K Alzubaidi, Ali Saleh A Alqarni

**Affiliations:** 1 Department of Anatomy, Umm Al-Qura University, Al-Qunfudah Medical College, Al-Qunfudah, SAU; 2 Department of Medicine and Surgery, Umm Al-Qura University, Al-Qunfudah Medical College, Al-Qunfudah, SAU; 3 Department of Mathematics, Umm Al-Qura University, Al-Qunfudah University College, Al-Qunfudah, SAU

**Keywords:** saudi arabia, southern region, western region, influencer, influence, rhinoplasty, adult, social media

## Abstract

Introduction

Rhinoplasty, a cosmetic surgical procedure aimed at altering the appearance of the nose, has gained immense popularity worldwide. Patients undergo this procedure for various reasons, ranging from aesthetic concerns to functional impairments. Social media, being a ubiquitous platform for sharing and consuming visual content, has emerged as a potential influencer for individuals contemplating rhinoplasty. This study aims to investigate the impact of social media on the prevalence of rhinoplasty among individuals residing in the southern and western regions of Saudi Arabia.

Methods

A cross-sectional study was conducted through an online self-administered questionnaire, targeting male and female adults aged 18 years or older, residing in the western and southern regions of Saudi Arabia. The questionnaire comprised 17 questions, categorized into two sections. The first section sought demographic information, including age, gender, education, and other relevant characteristics. The second section focused on the influence of social media on the decision-making process related to rhinoplasty.

Results

A total of 1645 participants responded to the survey, with 96.80% being Saudi citizens. The majority of respondents were females (69.11%); 58.52% of the respondents were from the western region of Saudi Arabia, while 41.48% lived in the southern region. Most participants (64.27%) were aged between 18 and 30 years. The study revealed that Snapchat (Snap Inc., Santa Monica, California, United States) was the most influential social media platform, with 43.41% of respondents reporting it as the key influencer for their decision to undergo rhinoplasty. Twitter (Twitter, Inc., San Francisco, California, United States) and Instagram (Meta Platforms, Inc., Menlo Park, California, United States) followed at 22.97% and 12.09%, respectively. Interestingly, 28.42% of respondents acknowledged that social media played a significant role in their decision to undergo rhinoplasty, particularly when promoted by celebrities or trusted figures. Comparing responses from the western and southern regions, the study showed that individuals from the southern region were relatively more influenced by social media, with 27.8% and 29.3% of respondents reporting the influence from the two regions, respectively. Out of the total respondents, only 38.75% reported dissatisfaction with their nose's appearance and condition, while 23.60% expressed a tendency towards undergoing rhinoplasty.

Conclusion

The study's findings underscore the critical role of social media in influencing patients' decisions to undergo rhinoplasty, particularly in the southern region of Saudi Arabia. Snapchat emerged as the most influential social media platform, with celebrities' pictures before and after the procedure being the leading factor in motivating patients to undergo rhinoplasty. The study highlights the need for further research to explore the potential risks and benefits associated with the influence of social media on patients' decision-making regarding rhinoplasty.

## Introduction

Rhinoplasty, a surgical procedure designed not only for cosmetic purposes but also to address nasal obstruction, is a meticulous surgery that demands precision and clinical skills to achieve optimal results [1]. The preoperative phase is crucial as it entails defining anatomical goals, exposing the deformity adequately, preserving normal anatomy, and restoring the nasal airway [2]. According to statistics, rhinoplasty accounted for the majority of the 2,314,720 cosmetic surgical procedures performed in 2020 with 352,555 procedures (15%) [3].

In Saudi Arabia, rhinoplasty constituted 60% of all plastic surgeries performed in 2019 [4]. A study conducted to determine the influence of social media on the eagerness to undergo facial cosmetic surgeries in Saudi Arabia revealed that 60.4% of participants believed that self-advertising surgeons played a critical role in the upsurge of cosmetic surgery trends, particularly among adolescents whose desire for cosmetic surgery, specifically rhinoplasty, is amplified by social media [5,6]. Two studies conducted in Riyadh [7] and Abha [8] reported that the use of celebrity pictures post-rhinoplasty on social media was the primary reason for the willingness to undergo the procedure.

Despite the growing popularity of cosmetic procedures and the increasing number of people desiring to undergo rhinoplasty, most studies conducted on the topic have focused primarily on central regions in Saudi Arabia. As such, the current study aims to examine the influence of social media platforms on rhinoplasty decisions in the western and southern regions of Saudi Arabia.

## Materials and methods

Sampling and data collection

This research utilized a cross-sectional design to investigate the influence of social media platforms on rhinoplasty decisions among adult residents of the western and southern regions of Saudi Arabia and compare the efficacy of the effect on enhancing or motivating the desire to undergo rhinoplastic surgery between the two different regions. The study included all individuals aged 18 years or older, irrespective of gender, who had not undergone rhinoplasty. It was conducted between September 22, 2022, and October 22, 2022. The sampling technique employed cluster sampling, whereby participants were randomly selected from two distinct regions and contacted through a WhatsApp broadcast message (Meta Platforms, Inc., Menlo Park, California, United States) that contained the questionnaire, study rationale, and research objectives. To ensure the survey's comprehensibility, 20 individuals familiar with the study's inclusion criteria, 10 from each region, pretested the questionnaire. The survey, which comprised 17 questions divided into two parts, was adapted from a previous study on social media's impact on rhinoplasty decisions [7]. The first part collected demographic data such as age, gender, marital status, education level, occupation, and region of residence from all participants. The second part included inquiries about social media use and its effect on rhinoplasty decisions. Participants gave written consent to participate, and their anonymity and data confidentiality was ensured.

Statistical analysis

In this study, IBM SPSS Statistics for Windows, Version 26.0 (Released 2019; IBM Corp., Armonk, New York, United States) was employed for data analysis. The results were presented using frequency and percentage figures for categorical data. The chi-square test was utilized to examine the association between demographic variables and selected variables, and a P-value of less than 0.05 was considered statistically significant. Furthermore, a comparative analysis of the results from the distinct regions was performed to identify the most influential factors in each region and evaluate their impact based on frequency and percentage.

Ethical consideration 

The Institutional Review Board of Umm Al-Qura University gave the approval for the study (approval number: HAPO-02-K-012-2022-11-1290).

## Results

In this study, a sample of 1682 participants responded to an online survey; 28 were excluded for being under the age of 18 years and the responses from 1654 were analyzed, predominantly comprising Saudi nationals (96.80%). The majority of respondents were female (69.11%), and their geographical distribution revealed a higher concentration in the western region of Saudi Arabia (58.52%), with the remaining respondents residing in the southern region (41.48%). Furthermore, most respondents were in the age group of 18-30 years (64.27%) and held a bachelor's degree (68.74%). Regarding their marital status, the sample consisted of a greater proportion of single individuals (48.25%) than married individuals (47.58%). Additionally, 48.31% of respondents were employed, while 32.29% were students. A comprehensive summary of the sample characteristics is presented in Table 1.

**Table 1:** Demographic Characteristics of Participants

				n		%	
1	Age (years)	18-30	1091		65.96%		
		31-40	295		17.84%		
		41-50	183		11.06%		
		51-60	75		4.53%		
		> 60	10		0.60%		
2	Sex	Female	1143		69.11%		
		Male	511		30.89%		
3	Nationality	Non -Saudi	53		3.20%		
		Saudi	1601		96.80%		
4	Social Situation	Divorced	52		3.14%		
		Married	787		47.58%		
		Single	798		48.25%		
		Widowed	17		1.03%		
5	Education Level	Elementary	17		1.03%		
		Middle School	27		1.63%		
		High School	265		16.02%		
		Diploma	94		5.68%		
		Bachelor	1137		68.74%		
		Master's	87		5.26%		
		PhD	27		1.63%		
6	Occupation	Student	534		32.29%		
		Employed	799		48.31%		
		Unemployed	281		16.99%		
		Retired	40		2.42%		
7	Region	Southern Region	686		41.48%		
		Western Region	968		58.52%		

**Figure 1:**
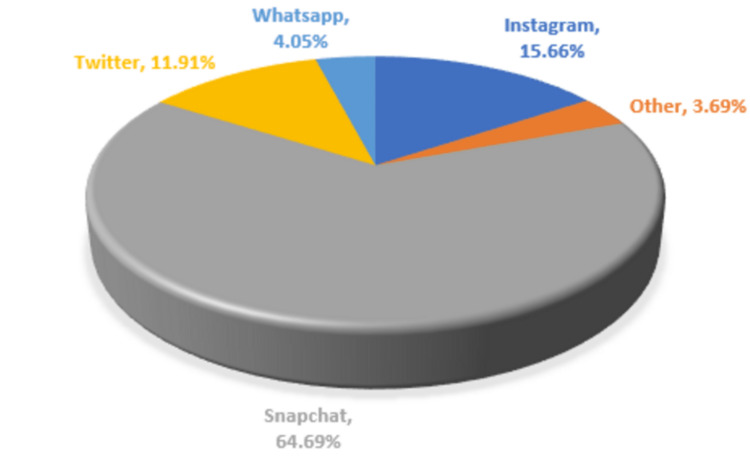
Most Common Social Media Platforms Used to Receive Information About Rhinoplasty Other: Google (Google LLC, Mountain View, California, United States), News, None, Reddit (Advance Publications, Inc., Staten Island, New York, United States), TikTok (ByteDance Ltd., Beijing, China), and YouTube (Google LLC)

The results indicate that Snapchat (Snap Inc., Santa Monica, California, United States) was the most frequently used social media platform (43.41%), followed by Twitter (22.97%) (Twitter, Inc., San Francisco, California, United States), while Instagram (Meta Platforms, Inc.) was the least used (12.09%). The analysis of social media use across demographic characteristics revealed that Snapchat use differed significantly across demographic characteristics except for gender (p=0.92945) (Table 2), whereas Instagram use varied significantly across demographic characteristics except for educational level (p=0.05297) (Table 3). Similarly, Twitter use differed significantly across various demographic characteristics except for nationality (p=0.47007) (Table 4), while WhatsApp use differed significantly across different social demographic characteristics except for nationality (p=0.05538) and region (p=0.05924) (Table 5). According to Figure 1, which presents data on the percentage of users who use various apps to find information about rhinoplasty, Snapchat is the most commonly used app with an overall percentage of 64.69%.

**Table 2:** Factors Associated With the Use of Snapchat* in Saudi Arabia *Snap Inc., Santa Monica, California, United States

		Snapchat Use		P-Value
		No (936)	Yes (718)	
Age (years)	18-30	595 (66.03%)	468 (65.87%)	0.00002
	31-40	143 (15.28%)	152 (21.17%)	
	41-50	118 (12.61%)	65 (9.05%)	
	51-60	47 (5.02%)	28 (3.90%)	
	> 60	10 (1.07%)	0 (0.00%)	
Sex	Female	646 (69.02%)	497 (69.22%)	0.92945
	Male	290 (30.98%)	221 (30.78%)	
Nationality	Non -Saudi	40 (4.27%)	13 (1.81%)	0.0048
	Saudi	896 (95.73%)	705 (98.19%)	
Social Situation	Divorced	24 (2.56%)	28 (3.90%)	< 0.00001
	Married	395 (42.20%)	392 (54.60%)	
	Single	504 (53.85%)	294 (40.95%)	
	Widowed	13 (1.39%)	4 (0.56%)	
Education Level	Elementary	15 (1.60%)	2 (0.28%)	0.00030
	Middle School	20 (2.14%)	7 (0.97%)	
	High School	169 (18.06%)	96 (13.37%)	
	Diploma	55 (5.88%)	39 (5.43%)	
	Bachelor	602 (64.32%)	535 (74.51%)	
	Master's	57 (6.09%)	30 (4.18%)	
	PhD	15 (1.60%)	9 (1.25%)	
Occupation	Student	378 (40.38%)	156 (21.73%)	< 0.00001
	Employed	385 (41.13%)	414 (57.66%)	
	Unemployed	146 (15.60%)	135 (18.80%)	
	Retired	27 (2.88%)	13 (1.81%)	
Region	Southern Region	351 (37.50%)	335 (46.66%)	0.00018
	Western Region	585 (62.50%)	383 (53.34%)	

**Table 3:** Factors Associated With the Use of Instagram* in Saudi Arabia *Meta Platforms, Inc., Menlo Park, California, United States

		Instagram Use		P-Value
		No (1454)	Yes (200)	
Age (years)	18-30	926 (64.99%)	146 (73.00%)	0.00423
	31-40	267 (18.36%)	28 (14.00%)	
	41-50	163 (11.21%)	20 (10.00%)	
	51-60	71 (4.88%)	4 (2.00%)	
	> 60	8 (0.55%)	2 (1.00%)	
Sex	Female	977 (67.19%)	166 (83.00%)	< 0.00001
	Male	477 (32.81%)	34 (17.00%)	
Nationality	Non -Saudi	40 (2.75%)	13 (6.50%)	0.00476
	Saudi	1414 (97.25%)	187 (93.50%)	
Social Situation	Divorced	46 (3.16%)	6 (3.00%)	0.00090
	Married	717 (49.31%)	70 (35.00%)	
	Single	675 (46.42%)	123 (61.50%)	
	Widowed	16 (1.10%)	1 (0.50%)	
Education Level	Elementary	13 (0.89%)	4 (2.00%)	0.05297
	Middle School	23 (1.58%)	4 (2.00%)	
	High School	225 (15.47%)	40 (20.00%)	
	Diploma	83 (5.71%)	11 (5.50%)	
	Bachelor	1001 (68.84%)	136 (68.00%)	
	Master's	85 (5.85%)	2 (1.00%)	
	PhD	24 (1.65%)	3 (1.50%)	
Occupation	Student	453 (31.16%)	81 (40.50%)	0.00001
	Employed	732 (50.34%)	67 (33.50%)	
	Unemployed	231 (15.89%)	50 (25.00%)	
	Retired	38 (2.61%)	2 (1.00%)	
Region	Southern Region	627 (43.12%)	59 (29.50%)	0.00025
	Western Region	827 (56.88%)	141 (70.50%)	

**Table 4:** Factors Associated With the Use of Twitter* in Saudi Arabia *Twitter, Inc., San Francisco, California, United States

		Twitter Use		P-Value
		No (1274)	Yes (380)	
Age (years)	18-30	782 (61.38%)	301 (81.31%)	< 0.00001
	31-40	246 (19.31%)	49 (12.89%)	
	41-50	167 (13.11%)	16 (4.21%)	
	51-60	70 (5.49%)	5 (1.32%)	
	> 60	9 (0.71%)	1 (0.26%)	
Sex	Female	902 (70.80%)	241 (63.42%)	0.00628
	Male	372 (29.20%)	139 (36.58%)	
Nationality	Non-Saudi	43 (3.38%)	10 (2.63%)	0.47007
	Saudi	1231 (96.62%)	370 (97.37%)	
Social Situation	Divorced	44 (3.45%)	8 (2.11%)	< 0.00001
	Married	657 (51.57%)	130 (34.21%)	
	Single	558 (43.80%)	240 (63.16%)	
	Widowed	15 (1.18%)	2 (0.53%)	
Education Level	Elementary	17 (1.33%)	0 (0.00%)	0.00012
	Middle School	23 (1.81%)	4 (1.05%)	
	High School	199 (15.62%)	66 (17.37%)	
	Diploma	74 (5.81%)	20 (5.26%)	
	Bachelor	888 (69.70%)	249 (65.53%)	
	Master's	50 (3.92%)	37 (9.74%)	
	PhD	23 (1.81%)	4 (1.05%)	
Occupation	Student	336 (26.37%)	198 (52.11%)	< 0.00001
	Employed	659 (51.73%)	140 (36.84%)	
	Unemployed	243 (19.07%)	38 (10.00%)	
	Retired	36 (2.83%)	4 (1.05%)	
Region	Southern Region	552 (43.33%)	134 (35.26%)	0.00510
	Western Region	722 (56.67%)	246 (64.74%)	

**Table 5:** Factors Associated With the use of WhatsApp* in Saudi Arabia. *Meta Platforms, Inc., Menlo Park, California, United States

		Whatsapp Use		P-Value
		No (1377)	Yes (277)	
Age (years)	18-30	999 (72.54%)	92 (33.21%)	< 0.00001
	31-40	235 (17.07%)	60 (21.66%)	
	41-50	103 (7.48%)	80 (28.88%)	
	51-60	37 (2.69%)	38 (13.72%)	
	> 60	3 (0.22%)	7 (2.53%)	
Sex	Female	968 (70.30%)	175 (63.18%)	0.01927
	Male	409 (29.70%)	102 (36.82%)	
Nationality	Non -Saudi	39 (2.83%)	14 (5.05%)	0.05538
	Saudi	1338 (97.17%)	263 (94.95%)	
Social Situation	Divorced	42 (3.05%)	10 (3.61%)	< 0.00001
	Married	601 (43.65%)	186 (67.15%)	
	Single	726 (52.72%)	72 (25.99%)	
	Widowed	8 (0.58%)	9 (3.25%)	
Education Level	Primary > Elementary	8 (0.58%)	9 (3.25%)	< 0.00001
	Medium > Middle School	18 (1.31%)	9 (3.25%)	
	High School	216 (15.69%)	49 (17.69%)	
	Diploma	70 (5.08%)	24 (8.66%)	
	Bachelor	978 (71.02%)	159 (57.40%)	
	Master's	69 (5.01%)	18 (6.50%)	
	PhD	18 (1.31%)	9 (3.25%)	
Occupation	Student	486 (35.29%)	48 (17.33%)	< 0.00001
	Employed	637 (46.26%)	162 (58.48%)	
	Unemployed	235 (17.07%)	46 (16.61%)	
	Retired	19 (1.38%)	21 (7.58%)	
Region	Southern Region	557 (40.45%)	129 (46.57%)	0.05924
	Western Region	820 (59.55%)	148 (53.43%)	

The findings of this study indicate that almost half of the respondents reported taking one to five selfies per day (46.92%) with a considerable proportion (28.42%) of the participants acknowledging the impact of social media on their decision to undergo rhinoplasty. It is noteworthy that a majority of participants reported rare encounters (35.55%) with news articles regarding rhinoplasty on social media platforms, while a significant proportion reported weekly exposure (33.19%). The results also revealed that Snapchat was the most frequently used platform for accessing articles about rhinoplasty (64.69%). Furthermore, nearly one-third of the respondents (29.9%) expressed belief in the safety of rhinoplasty based on social media advertisements, while a quarter (25.21%) reported being influenced by celebrity images before and after the procedure. Notably, a substantial proportion of participants (30.89%) reported being influenced by advertisements promoting non-surgical cosmetic procedures, followed by those promoting surgical cosmetic procedures (22.97%). In terms of participant satisfaction with the shape of their noses, a majority (61.25%) reported contentment, while a significant proportion (38.75%) expressed dissatisfaction. Among those dissatisfied, a considerable proportion (23.60%) expressed a desire to undergo rhinoplastic surgery. The reasons for considering surgery were primarily for the purpose of enhancing self-confidence (32.48%) or improving appearance (32.48%). On the other hand, the majority (76.36%) of participants reported no intention to undergo rhinoplastic surgery, citing reasons such as self-acceptance and the belief that cosmetic surgeries do not offer any benefits (67.70%). These findings are presented in Table 6.

**Table 6:** Participants’ Perceptions and Social Media Advertisements (n = 1654)

	Category	Frequency		%
Has social media affected you to undergo nose cosmetics?	No	1184	71.58%	
	Yes	470	28.42%	
How many times do you find news articles about rhinoplasty on social media?	Rarely	588	35.55%	
	Daily	190	11.49%	
	Weekly	549	33.19%	
	Monthly	327	19.77%	
Do you believe ads that provide rhinoplasty as a safe surgical procedure?	No	1209	73.10%	
	Yes	445	26.90%	
Have you been affected by a celebrity picture before and after rhinoplasty ?	No	1237	74.79%	
	Yes	417	25.21%	
What kind of cosmetic treatment are you getting influenced toward by advertisements on social media?	Non-surgical	511	30.89%	
	Surgical	380	22.97%	
	Both	311	18.80%	
	No, I am not affected	452	27.33%	
How do you feel about the appearance of your nose?	happy	1013	61.25%	
	Not happy	641	38.75%	
Do you want to do a plastic surgery for your nose?	No	1263	76.36%	
	Yes	391	23.64%	
How many times are you taking pictures (selfies) daily?	1-5 times	776	46.92%	
	6-10 times	207	12.52%	
	> 10 times	32	1.93%	
	I don't take selfies	639	38.63%	
Why do you want to do a nose cosmetic?	Better appearance	127	32.48%	
	Correction of a defect and curvataure	31	7.93%	
	Fashion follower	86	21.99%	
	For myself	12	3.07%	
	Not satisfied with shape	8	2.05%	
	To become more confident	127	32.48%	
Why do you not want to do a nose cosmetic?	Fear of side effects	191	15.12%	
	I admire myself and there is no need for plastic surgery	855	67.70%	
	I do not have enough money	148	11.72%	
	Religious reasons	41	3.25%	
	Satisfaction	28	2.22%	

The analysis of the data revealed a statistically significant association between the frequency of taking selfies and the inclination towards rhinoplastic surgery, with a p-value of 0.000. Specifically, the findings indicated that participants who take a higher number of selfies per day were more likely to express a desire for rhinoplastic surgery. Notably, a substantial proportion (66.2%) of participants who reported taking 6-10 selfies per day expressed a desire for rhinoplasty. Further details on this association can be found in Table 7.

**Table 7:** Relationship Between Taking Selfies and the Desire to Undergo Rhinoplasty

	Do you want to do a plastic surgery for your nose?		
How many times are you taking pictures (selfies) daily?	Yes	No	P-Value
I don't take selfies	66 (10.3%)	573 (89.7%)	0.000
1-5 times	178 (22.9%)	598 (77.1%)	0.000
6-10 times	137 (66.2%)	70 (33.8%)	0.000
More than 10 times	10 (31.1%)	22 (68.8%)	0.000

Table 8 displays the results of a study that examined the influence of social media and celebrity photos on individuals' decisions to undergo rhinoplasty, as well as their beliefs and attitudes toward the safety of this procedure. The study found that participants from the southern region were more likely to be influenced by social media, with 29.3% of participants from this region reporting that social media played a role in their decision to undergo rhinoplasty. In contrast, participants from the western region were less likely to be influenced by social media, with only 27.8% of participants reporting such influence.

**Table 8:** Comparison Between Western and Southern Regions on the Influence of Social Media on Rhinoplasty Decision

	Southern region (686)		Western region (968)	
	Yes	No	Yes	No
Has social media influenced you to undergo rhinoplasty	201 (29.3%)	485 (70.7%)	269 (27.8%)	699(72.2%)
Have you been affected by a celebrity picture before and after rhinoplasty?	185 (27.0%)	501 (73.0%)	232 (24.0%)	736 (76.0%)
Do you believe advertisements that promote rhinoplasty as a safe procedure?	222 (32.4%)	464 (67.6%)	223 (23.0%)	745 (77.0%)
Do you want to undergo rhinoplasty?	172 (25.1%)	514 (74.9%)	219 (22.19%)	749 (77.4%)

Moreover, when it comes to the impact of celebrity photos, the study revealed that participants from the southern region were more affected, with 27% of them reporting that celebrity photos before and after rhinoplasty influenced their decision, compared to 24% of participants from the western region. The study also found that participants from the southern region were more likely to believe that rhinoplasty is a safe procedure, with 32.4% of them holding this belief, while only 23% of participants from the western region believed so.

Tables 8-9 present additional findings that indicate differences between the southern and western regions in terms of the availability of information about rhinoplasty and individuals' desire to undergo this procedure. According to Table 9, a higher percentage of participants from the southern region reported finding articles about rhinoplasty weekly (33.5%) and monthly (21.9%), while a higher percentage of participants (37.2%) from the western region reported rarely finding new articles about rhinoplasty. Additionally, the desire to undergo rhinoplasty was found to be higher among participants from the southern region (25.1%) compared to those from the western region (22.19%).

**Table 9:** Rate of Finding Articles About Rhinoplasty in the Two Regions

	Southern region (n=686)				Western region (n=968)				
	Daily	Weekly	Monthly	Rarely	Daily	Weekly	Monthly	Rarely	
How frequently do you find articles about rhinoplasty?	78 (11.4%)	230 (33.5%)	150 (21.9%)	228 (33.2%)	112 (11.6%)	319 (33.0%)	177 (18.3%)	360 (37.2%)	

The present study aimed to investigate the relationship between individuals' beliefs about the safety of rhinoplasty and their desire to undergo the procedure. The results of the study revealed a significant relationship between these two variables, with a p-value of 0.000, indicating that those who believed rhinoplasty to be a safe procedure were more likely to express a desire to undergo it.

To further explore regional differences in this relationship, the study examined the relationship between believing rhinoplasty to be safe and the desire to undergo it among participants from the southern and western regions. Table 10 presents the findings of this analysis, indicating that participants from the southern region who believed the procedure to be safe were more willing to undergo it, with 78.5% of them expressing a desire to do so. In contrast, among participants from the western region who believed the procedure to be safe, 64.6% expressed a desire to undergo it.

**Table 10:** Relationship between believing in Advertisements Promoting Rhinoplasty as a Safe Procedure and the Desire to Undergo Rhinoplasty in the Western and Southern Regions

	Do you believe advertisments that promote rhinoplasty as a safe surgical procedure?				
	Southern Region (n=686)		Western Region (n=968)		
Do you want a plastic surgery for your nose?	Yes	No	Yes	No	P-Value
Yes	135 (78.5%)	37 (21.5%)	141 (64.6%)	78 (35.6%)	0.000
No	87 (16.9%)	427 (83.1%)	82 (10.9%)	676 (89.1%)	0.000

## Discussion

This study was conducted to investigate the effect of social media on individuals from western and southern regions of Saudi Arabia. According to a study conducted to measure the social media effect to induce eagerness to perform facial cosmetic surgeries in Saudi Arabia, 60,4% of the partakers agreed that self-advertising surgeons played a crucial role in the growth of the cosmetic surgery trend (5). Also, according to a study conducted previously in the central region of Saudi Arabia among patients who underwent rhinoplasty, most of the study sample were influenced by celebrity pictures before and after [7]. Hence, this study aimed to examine and highlight the influential role of social media on the proliferation of rhinoplasty procedures in the western and southern regions.

A total of 1654 participants in this study completed the online questionnaire. The main finding is that 28.42% of participants declared that they have been influenced by social media, mainly by pictures of celebrities before and after rhinoplasty; 64.69% reported that they find most of the articles and pictures about rhinoplasty on Snapchat and 46.92% stated that they take around one to five selfies per day. Around 23.6% admitted that they desired to undergo rhinoplasty either to have a better appearance or to become more confident. As the results revealed, the participants from the southern region showed more possibility of being influenced by social media to decide to undergo rhinoplasty than their peers in the western region.

A similar study about social media and its effect on undergoing rhinoplasty was conducted in Riyadh, in which 205 participants who underwent rhinoplasty in the private sector completed an online questionnaire [7]. The study stated that 52.7% of respondents were influenced by social media. In the current study, only 28.4% of participants were influenced by social media. The reason behind the difference between the results could be due to different study samples as the current study was among individuals who had not undergone rhinoplasty while Obeid et al.'s study included a population who had already done rhinoplasty.

While Snapchat was the most used platform, in the studies by Obeid et al. [7] and Alghamdi et al. [8], the participants reported that they were mainly influenced by after pictures of rhinoplastic procedures. Both findings align with the findings of the present study as Snapchat was the most used social media platform (64.69%) and pictures of celebrity before and after rhinoplasty was the most reported reason to undergo rhinoplasty. This could be a result of facilitated accessibility to Snapchat as one of its features is to recommend influencers' accounts where most of them are posting the results after doing the procedure [9-11].

In another study conducted in Aseer Central Hospital to assess the role of social media to perform rhinoplasty, 100 patients who underwent rhinoplasty between 2015 and 2020 filled out a pre-structured questionnaire through their mobile phones. Their findings suggest that one-third of respondents browsed social media to explore rhinoplasty on a monthly basis. In the current study, respondents from the western region reported rarely finding articles regarding rhinoplasty through social media (37.2%). Interestingly, participants from the southern region find articles regarding rhinoplasty in social media weekly (33.5%). Surprisingly the participants from the southern region showed more interest to perform rhinoplasty. The reason behind that difference could be due to the reduced availability of cosmetic procedure services in the southern region which increases the attraction to perform cosmetic procedures, while in the western region, the accessibility to plastic surgery services is easier [12-13]. Blau et.al. conducted a study in the United States to assess the public demand for plastic surgery in different states based on Google search and their findings were suggestive of the increased demand for this kind of surgery in areas with low number of plastic surgeons [12]. According to the Ministry of Health (MOH) annual statistics, they reported that the health sectors in the southern region have fewer plastic surgeons in comparison with the western region [13].

A study conducted to investigate the reasons behind the trending of facial plastic surgery suggested that non-surgical procedures are more in demand than surgical, as Botox was the most desired intervention while rhinoplasty was the most demanded surgical procedure [4]. In the present study too, the participants showed more inclination toward non-surgical procedures than surgical.

According to a study conducted by Aldosari et al. to assess the influence of media on facial plastic surgery, there is increased urge to perform rhinoplasty to look better in selfies [5]. This aligns with the current study findings as the participants who desired to undergo rhinoplasty declared that the reason behind their desire is to have a better appearance and the participants who took more selfies showed more affiliation to rhinoplasty. This could be a result of coronavirus disease 2019 (COVID-19) pandemic as it led to increased utilization of video conferencing platforms showing only the face and its characteristics. Also, the American Academy of Facial Plastic and Reconstructive Surgery (AAFPRS) annual survey found that 79% of facial plastic surgeons reported an increased demand in facial cosmetic to look better or to have a better pictures [14]. Furthermore, 38.75% of the participants in the current study were bothered by the shape of their noses. This is in line with the findings of Mianroodi et al.'s study conducted to investigate the interest of female high school students in rhinoplasty and assess their awareness about their postoperative complications, which found that 37.7% of their participants were not happy with the shape of their noses [15].

Recommendations

This study represents the first of its kind in the Kingdom of Saudi Arabia, examining the comparative influence of social media on individuals seeking rhinoplasty in two distinct regions. The results of this study underscore the need to raise awareness among the general populace regarding the appropriate indications for rhinoplasty and to utilize social media as a means of disseminating evidence-based information on this subject. Furthermore, this study highlights the necessity of augmenting the number of plastic and facial plastic surgeons practicing in the southern region.

Limitations

However, it is important to acknowledge several limitations of the current study. Firstly, our participant pool was drawn from a relatively narrow social demographic group, which may limit the generalizability of our findings. Additionally, we encountered challenges in accessing facial plastic surgery clinics and were only able to survey participants from the nearest regions, thus restricting our ability to gather data from other regions of Saudi Arabia. Finally, we faced difficulties in accessing patients who had already undergone rhinoplasty, further constraining our sample size.

## Conclusions

The present study revealed that social media platforms play a significant role in the decision-making process of individuals seeking rhinoplasty. Notably, a majority of participants reported frequent use of Snapchat, and identified this platform as the primary source of information and visual content related to the procedure. The prominent influence of celebrity photographs depicting successful outcomes of rhinoplasty was identified as the main motivator for participants considering the surgery, with the primary goal being enhanced physical appearance and increased self-confidence. Regional differences were also observed, with participants from the southern region displaying a higher susceptibility to social media influences, as compared to those residing in the western region. This difference was attributed to the greater visibility of celebrity rhinoplasty success stories in the southern region, leading to the perception of rhinoplasty as a safe and attainable cosmetic option, further reinforced by the abundance of information and imagery on social media. Ultimately, these findings suggest a heightened desire for rhinoplasty among individuals in the southern region, largely driven by the pervasive influence of social media.
